# Targeted Therapy in Non-Muscle Invasive Bladder Cancer-Clinical Updates

**DOI:** 10.33696/pharmacol.6.056

**Published:** 2024

**Authors:** Rachel Passarelli, John Pfail, David Golombos, Thomas L. Jang, Vignesh T. Packiam, Saum Ghodoussipour

**Affiliations:** 1 Division of Urology, Rutgers Robert Wood Johnson University Hospital, New Brunswick, NJ 08901, USA; 2 Section of Urologic Oncology, Rutgers Cancer Institute of New Jersey, New Brunswick, NJ 08901, USA

**Keywords:** Bladder cancer, Target therapy, Precision medicine, Non muscle invasive bladder cancer

## Abstract

Non muscle invasive bladder cancer (NMIBC) comprises almost 75% of all bladder cancer (BC) diagnoses. Longstanding intravesical treatment options include chemotherapy or Bacillus Calmette-Guerin (BCG). However, despite these treatment options, there is a high rate of relapse for NMIBC patients, reaching up to 40–50% for patients with high risk disease. Radical cystectomy is recommended by guideline committees for patients with high risk NMIBC and for patients who fail intravesical treatment options however this is a procedure with high morbidity and many patients are not good candidates or prefer more conservative treatment approaches. Given this and the high failure rates of current therapies, much ongoing research in NMIBC is focused on other bladder sparing treatment modalities. There have been new advances in molecular features of BC with findings of genetic alterations in NMIBC leading way to a rise in precision medicine targeting a patient’s individual gene mutation. While data surrounding these targeted therapies in NMIBC specifically are all preliminary, many trials are currently underway derived from success in targeted treatments for locally advanced or metastatic BC. Preliminary results are promising and the targeted therapies for NMIBC are increasing. We performed a PubMed and Medline (OVID) literature review assessing recently published studies on targeted therapy on NMIBC, and an additional search on clinicaltrials.gov for active clinical trials. We report on preliminary outcomes and ongoing later stage trials for targeted therapies in NMIBC and anticipate upcoming promising changes to the treatment landscape.

## Introduction

Bladder cancer (BC) is predicted to be the fourth most diagnosed cancer in men and eleventh in women in 2024 with an estimated 83,000 new diagnoses [1]. Diagnosis is made based on grade and stage following resection, and patients are further divided into subgroups- muscle invasive bladder cancer (MIBC) and non-muscle invasive bladder cancer (NMIBC). NMIBC encompasses almost 75% of BC diagnoses. Guideline based treatment for NMIBC consists of cystoscopy and complete endoscopic resection followed by observation, intravesical chemotherapy or Bacillus Calmette-Guerin (BCG) based on risk stratification [2–4]. Among patients with high-risk (HR) NMIBC in particular, there is a high relapse rate for intravesical treatment, up to 40–50% [5,6].

American Urological Association guidelines support radical cystectomy (RC) as an option for certain patients with HR NMIBC and those who fail intravesical treatment options [2]. However, RC is associated with significant post operative morbidity and not all patients are fit candidates, or may prefer bladder sparing treatment strategies. Given the high failure and recurrence rates of current treatment options and the morbidity associated with RC, ongoing research in the NMIBC community focuses on treatment options that can delay or avoid RC. Furthermore, the BCG shortage has led to a decrease of patients with intermediate risk (IR) NMIBC receiving this treatment, and the search continues for an effective, well-tolerated, and available therapy for this cohort of patients.

There have been many recent advances in understanding the molecular features of bladder cancer with findings of genetic alterations in FGFR3, PIK3CA, STAG2, and RTK/RAS/RAF genes in NMIBC [7]. Exploration of these pathways allowed for the potential for precision medicine, where therapies can be selected to target a patient’s individual gene mutation. There is currently little published data on targeted therapy for NMIBC, however many current trials are underway that originated from success in targeted treatments for locally advanced or metastatic BC. While none of these medications are yet FDA approved for NMIBC treatment, preliminary results are promising and trials assessing targeted therapy options are increasing with time. These therapies are primarily studied as salvage treatments for patients in whom BCG fails, but may be considered as up-front alternatives to BCG in the future. We aim to provide a narrative review of current literature assessing targeted therapy options and ongoing clinical trials for NMIBC.

## Methods

A literature search was conducted of PubMed and Medline (OVID) identifying recently published studies on targeted therapy for NMIBC. An additional search was performed via clinicaltrials.gov to find active clinical trials on target therapy for NMIBC (Table 1). We searched for recent conference presentations from these active clinical trials. Search terms included NMIBC, targeted therapy, epithelial protein markers, fibroblast growth factor (FGFR), vascular endothelial growth factor (VEGF) and selective replication viral vectors. We included all articles and presentations that were available in English.

## Discussion

### Epithelial surface receptor targets

#### Vicinium:

Oportuzumab monatox (Vicinium), a recombinant fusion protein containing an anti-epithelial cell adhesion molecule (epCAM) antibody that is linked to *Pseudomonas* exotoxin A (ETA), has been studied as an intravesical option for NMIBC. A Phase 2 study was conducted assessing efficacy of Vicinium in patients with carcinoma in situ (CIS) who had previously failed BCG therapy. A total of 46 patients received either one 6-week induction cycle followed by up to 3 maintenance cycles of 3 weekly administrations every 3 months (cohort 1) or one 12-week induction cycle with the aforementioned maintenance schedule (cohort 2). Of these patients, 9 (41%) in cohort 1 and 9 (39%) in cohort 2 achieved complete response (CR) at the 3-month evaluation. Median time to recurrence was 274 days and 408 days respectively in cohorts 1 and 2. There were 7 patients overall (16%) who remained disease free at last follow up (18–25 months) [8]. A Phase 3 trial presented at the 2020 AUA annual meeting assessed response to Vicinium in BCG unresponsive patients and demonstrated promising results. All patients in this trial received twice weekly intravesical Vicinium instillations for 6 weeks, followed by weekly instillations for another 6 weeks with further maintenance therapy depending on disease status. Of the 89 patients with CIS, 40% achieved CR at initial 3 months follow up, and 52% of initial responders maintained CR at 12 months. For patients with papillary disease, of which there were 38, CR at 3 and 12 months was 71% and 50% respectively. There were 10% of patients (6/63) of the initial 3-month responders who progressed to RC compared to 32% (18/56) of initial non-responders. There were 4 treatment related adverse events(AE) noted in 3 patients including grade 4 cholestatic hepatitis, grade 5 renal failure, grade 3 acute kidney injury, and grade 2 pyrexia [9]. Taken together, these two studies suggest that Vicinium can be a well-tolerated option for patients with BCG unresponsive disease, and may prove to aid in delaying RC.

In addition to monotherapy, Vicinium has also been investigated as a combination therapy. An interim analysis of a Phase 1 study of Vicinium and Durvalumab (a PD-L1 inhibitor) in 12 patients with high grade (HG) NMIBC previously treated with BCG yielded a disease free rate of 42% at first 12 week evaluation, 33% at 6 months, and 17% at 12 months with only 1 grade 3 or higher AE [10].

#### Enfortumab vedotin :

Enfortumab vedotin (EV), a drug antibody complex directed against nectin-4 shown to be highly expressed in urothelial carcinoma (UC), was FDA approved in late 2023 as a combination therapy approach with Pembrolizumab for patients with locally advanced or metastatic BC [11]. A recent Phase 3 clinical trial, EV-301, assessed response to EV versus chemotherapy in patients with locally advanced or metastatic UC and progression after platinum-based chemotherapy and PD-1/PD-L1 inhibitor treatment. In this trial of 608 patients, risk of death was reduced by 30% in the EV arm compared to chemotherapy (hazard ratio (HR) 0.70, 95% confidence interval [CI] 0.58–0.85) [12]. Based on preliminary results from the aforementioned trial, EV has also been investigated as an intravesical treatment for patients with NMIBC. EV-104 (NCT05014139) is a currently ongoing clinical trial assessing safety and response to intravesical EV in patients with BCG unresponsive NMIBC who are not eligible for or refuse RC. Preliminary data of 6 patients at various doses is promising with no Grade 3 or higher AE and 3/4 CR in patients undergoing the lower dose regimen. Response has not been assessed yet in the higher doses [13,14].

#### Oncofid-P-B:

Oncofib-P-B is a novel agent consisting of paclitaxel conjugated with hyaluronic acid (HA). Paclitaxel is a taxane agent that interferes with microtubule disassembly similar to docetaxel. Compared to docetaxel, paclitaxel is poorly soluble in water and thus if not prepared with appropriate co-agents, is less effective. Cremopher, a non-ionic surfactant can be utilized in preparation of paclitaxel to increase water solubility. However, it can induce histamine release causing irritation and reactions as well as induce the formation of micelles that limit paclitaxel delivery when administered intravesically [15,16]. The combination of paclitaxel and HA in Oncofib-P-B serves to improve the water solubility of paclitaxel, increasing local activity by allowing the binding of the HA moiety to CD44 receptors that are overexpressed on the surface of urothelial tumors.

A multicenter Phase 1 study assessed the safety and tolerability of intravesical Oncofid-P-B in 20 patients with BCG unresponsive NMIBC with or without Ta/T1 disease. Following the initial 12 week treatment doses, CR was achieved in 75% (15/20) of patients, with CR maintained in 13(65%), 12 (60%), 9 (45%) and 8 (40%) at 3, 6, 9 and 12 months respectively. Treatment was well tolerated without any severe AE and no drug related treatment discontinuation [17]. Oncofid-P-B is currently under investigation for patients with BCG unresponsive CIS with or without papillary disease in a Phase 3 trial (NCT05024773).

### Fibroblast growth factor pathway

#### Erdafitinib:

Erdafitinib, a pan-FGFR tyrosine kinase inhibitor (TKI), was initially FDA approved in 2019 for use in a specific subset of patients with locally advanced or metastatic UC harboring relevant FGFR3 or FGFR2 genetic alterations who progressed despite platinum chemotherapy treatment [18]. This was based on a study by Loriot *et al*. who showed that in a subset of 99 patients with either locally advanced, unresectable or metastatic UC with disease progression despite platinum chemotherapy, 40% of patients achieved a confirmed response with Erdafitnib. Progression-free survival (PFS) was noted to be 5.5 months, and the median duration of overall survival (OS) was 13.8 months. However, dose related AE were high, with grade 3 or higher AE occurring in 46% of patients and a discontinuation rate of 13% [19]. Guercio *et al*. evaluated patients with UC for FGFR3 alterations predictive of Erdafitinib sensitivity finding 14% of patients with MIBC, 39% of patients with NMIBC, 43% of patients with localized upper tract urothelial carcinoma (UTUC), and 26% of patients with metastatic disease harbored these mutations. On their evaluation, there was significant dose related toxicity, and median PFS and OS were 2.8 and 6.6 months respectively, although 40% of patients had some response [20].

Erdafitinib has been studied specifically in NMIBC patients with preliminarily positive results. FGFR alterations are shown to be even more common in NMIBC, up to 60–70% compared to 15–20% of patients with metastatic disease [21,22]. Catto et al. recently published on Cohort 2 of the THOR-2 study, which assessed patients with papillary only HG T1/Ta recurrence after BCG treatment with select FGFR mutations in their response to oral Erdafitinib for 6 weeks compared to intravesical chemotherapy with either single agent mitomycin C or gemcitabine. After accruing 77 patients with a median follow up of 13.4 months, median recurrence free survival (RFS) was not reached in patients in the Erdafitinib arm compared to 11.6 months in the intravesical chemotherapy arm, with an estimated HR of 0.28 (95% CI 0.1–0.6). Safety of drug administration was consistent with prior studies [23]. Cohort 3 of this study was recently presented by Daneshmand et al. and assessed Erdafitinib use in patients with recurrent IR low grade (LG) Ta/T1 NMIBC and FGFR alterations (NCT04172675). Patients underwent TURBT leaving behind a marker lesion (5–10 mm) and subsequently received daily oral Erdafitinib for 28 days. At data cut off, out of 10 patients, 6 had achieved CR and 1 achieved partial response with median duration of response 2.8 months. The two most common AE were hyperphosphatemia which occurred in 90% of patients and diarrhea in 60% [24].

#### TAR 210:

In addition to oral treatment, Erdafitinib has been investigated as an intravesical treatment option with aim to minimize systemic absorption and increase tolerability. TAR-210 is an intravesical drug delivery system with that provides local continuous release of Erdafitinib within the bladder for 90 days. TAR-210 is currently undergoing evaluation in a variety of NMIBC patients- those with papillary HR NMIBC who have recurred after BCG and are ineligible for, refusing or scheduled for RC, patients with IR recurrent Ta/T1 with LG disease, and patients with MIBC who are ineligible or refusing RC and cisplatin chemotherapy (NCT05316155). A Phase 1 study (NCT05316155) evaluated safety, pharmacokinetics, and response of TAR-210 in patients with NMIBC and FGFR alterations. At the time of data presentation, 6 patients with BCG refractory and 27 patients with IR disease were examined with a CR of 82% and 87% respectively. There were two patients who discontinued treatment secondary to urinary symptoms and one patient had a serious AE of pyelonephritis and sepsis [25,26]. A Phase 3 trial (MoonRISe-1, NCT06319820) is currently underway specifically examining TAR-210 in patients with IR NMIBC and FGFR alterations.

#### Pemigatinib:

Pemigatinib, a selective oral inhibitor of FGFR1-3 has also been investigated as a treatment option in UC. The FIGHT-201 trial assessed efficacy and safety of Pemigatinib in patients with FGFR3 mutations (cohort A) or FGF/FGFR alterations (cohort B) who had previously treated, unresectable or metastatic UC. The study enrolled a total of 260 patients and noted an objective response rate of 17.8% and 23.3%, respectively in two cohorts of patients who received continuous dosing or intermittent dosing. Furthermore, median duration of response was 6.2, 6.2 months and OS was 6.8, 8.9 months in the continuous and intermittent dosed cohorts, respectively. Pemigatinib was not noted to be efficacious in cohort B [27]. There is a current ongoing Phase 2 clinical trial (NCT03914794) assessing response to Pemigatinib before TURBT in patients who have recurrent tumors with a history of previous low risk (LR) or IR NMIBC.

### Vascular endothelial growth factor receptor pathway

#### Sunitinib and Dovitinib:

Targeting the VEGF pathway has long been shown to be a potential target for UC through inhibiting mitogenic and angiogenic processes [28]. Additionally, elevated VEGF levels have been shown to be markers of more aggressive disease and worse outcomes for patients [29]. Initial studies investigated Sunitinib and Pazopanib, VEGF TKIs in the metastatic UC setting which yielded low response rates and high overall toxicity [30,31]. Cabozantinib, an alternative TKI has also been investigated in the metastatic UC space with more promising results. The ATLANTIS trial, a Phase 2 study that assessed response to cabozantinib in patients with metastatic UC who had previously shown halted progression with platinum based therapy, identified no benefit compared to placebo in these patients [32]. The COSMIC-021 study assessed combination therapy with cabozantinib and atezolizumab in three cohorts of patients- those prior chemotherapy naïve but platinum ineligible (cohort 3), prior chemotherapy naïve but platinum eligible (cohort 4), and those who had received prior immune checkpoint inhibitor therapy (cohort 5). Of these patients, disease control rates were noted to be 80%, 63%, 61% in cohorts 3, 4, and 5 respectively with manageable toxicity [33].

For NMIBC specifically, Sunitinib and Dovitinib have been investigated as options for BCG unresponsive patients. In a Phase 2 single arm study, Zahoor et al. evaluated response to Sunitinib in patients with BCG refractory NMIBC and hypothesized that this drug may decrease progression or recurrence. They found that 15/19 (79%) patients were able to tolerate the complete treatment course while four patients discontinued because of AE. At twelve weeks, 8/18 (44%) maintained CR, 9/18(50%) experienced progression and 1/18(5.6%) recurrence. Of those that initially had a response, 4 patients (22% overall) were progression free at 12 months. Overall, the authors concluded that sunitinib was not associated with improved clinical outcomes in BCG refractory NMIBC [34]. Dovitinib, a TKI of both FGFR1-3, VEGFR1-3, has also been studied as an option for BCG unresponsive NMIBC. In a multicenter Phase 2 trial, patients with BCG unresponsive NMIBC with increased FGFR3 expression or FGFR3 mutations received oral Dovitinib. At the 6-month TURBT mark, of 13 patients, 8% had CR, however pharmacologically active forms of the drug were present in the urothelial tissue of all patients and post treatment staining showed reduced pFGFR3. However, toxicity was very high with all patients experiencing at least one Grade 3 or 4 AE and this study was ultimately terminated as development of Dovitinib was halted [35].

### Cretostimogene grenadenorepvec

Cretostimogene grenadenorepvec (CG) formerly known as CG0070, is an oncolytic adenovirus that selectively replicates in retinoblastoma (RB) pathway defective bladder tumor cells. CG has been previously shown to be a safe and effective treatment for patients with BCG unresponsive NMIBC, and was approved for fast track designation for this cohort of patients in late 2023. Initially studied by Burk *et al*. in a Phase 1 study of patients with NMIBC who had failed at least one course of BCG, a CR of 48.6% over median duration of 10.4 months was noted. Authors found an increased CR rate, up to 81.8% in patients with higher RB pathway alterations [36]. In a study of 45 BCG unresponsive patients who underwent intravesical CG treatment monotherapy, overall CR at 6 months was noted to be 47%. When stratified by disease status, CR was noted to be 58% in patients with pure CIS, 50% for CIS ± Ta/T1, and 33% for pure Ta/T1 [37]. BOND-003(NCT04452591) is a Phase 3 trial assessing intravesical CG response in patients with completely resected BCG unresponsive HR NMIBC unwilling or unfit for RC. Initial results of 66 patients suggested an anytime CR of 75.7% with 74.4% CR maintained at >6 months. Of patients who initially did not respond, 31% were able to be salvaged with re-induction. Only Grade 1 and 2 AE were reported, and there were no AE related treatment discontinuations [38]. Recently launched is the PIVOT-006 (NCT06111235) Phase 3 randomized study of adjuvant CG compared to observation for IR NMIBC patients. CG has further been investigated in combinations with other therapies. Preliminary results of a combination approach with intravesical CG and IV pembrolizumab in a cohort of 24 patients with BCG unresponsive NMBIC have shown promising results of 92% CR at 3 months, and 75% at 1 year for the 8 patient sub-cohort with sufficient follow-up [39].

## Conclusions

Given high recurrence rates of NMIBC there have been efforts to investigate alternative treatment options to the historical gold standard, BCG. A rise in precision medicine and targeting mutations in specific genetic pathways in cancer treatment has also been prevalent in bladder cancer research. Most published studies about targeted approaches have focused on locally advanced and metastatic disease, however promising outcomes have allowed extrapolation induced studies focusing on NMIBC. Given the early stage at which research into these treatment options for NMIBC stands, there are no targeted therapies for NMIBC that are yet FDA approved. However, the preliminary data that has been accrued is promising and as studies continue more options will be available and formally approved for patients. While many of the discussed options in this review are targeted therapies, there are few reliable biomarkers that exist outside of FGFR testing to aid in patient specific treatment choice. This is an area of active research, and in the future, we anticipate there will be tissue and urinary biomarkers that are readily available for this purpose. In this review, we report on predominately preliminary results of early trials investigating targeted techniques in NMIBC treatment, as well as discussion of currently ongoing later stage trials with promising results on the horizon.

## Figures and Tables

**Table 1.** Active target therapy clinical trials in NMIBC.

Study	Phase	Agent	Primary Endpoint	Estimated Primary Completion Date	Population
NCT04172675	II	Erdafitinib versus Intravesical Chemotherapy	Recurrence free survival	2024-03-29	HR NMIBC and FGFR mutations or fusions with recurrence after BCG
NCT05316155	I	Erdafitinib intravesical delivery system	Determine dose and preliminary clinical efficacy	2028-03-30	NMIBC or MIBC patients with FGFR mutations/fusions
NCT05567185	I	Erdafitinib Intravesical Delivery System	Tolerability	2026-10-16	Bladder Cancer and Selected FGFR Mutations or Fusions
NCT04917809	II	Oral Erdafitinib	Objective response rate	2025-08	Recurrent NMIBC with FGFR mutations
NCT06319820 (MoonRISe-1)	III	TAR-210 vs intravesical chemotherapy	Disease free survival	2028-06-28	intermediate-risk NMIBC and FGFR mutations
NCT03914794	II	Pemigatinib prior to TURBT	Complete Response	2024-05	LR or IR NMIBC Recurrence
NCT05014139	I	Intravesical Enfortumab Vedotin	Incidence of AE, dose limiting toxicities, laboratory anomalies	2024-06-30	BCG unresponsive CIS with or without papillary NMIBC
NCT06111235	III	CG after TURBT vs surveillance	Recurrence Free Survival	2028-01	IR-NMIBC
NCT06253845 (PIVOT-006)	I	Intravesical CG	Incidence of Treatment Emergent AE	2027-02	IR-NMIBC
NCT04452591	III	Intravesical CG	Complete response	2024-01-31	BCG unresponsive CIS with or without HG NMIBC
NCT04387461 (CORE-001)	II	Intravesical CG + IV Pembrolizumab	CR	2023-06	BCG unresponsive CIS with or without HG NMIBC
NCT05024773	III	Oncofid-P-B	CR	2027-11	BCG unresponsive CIS with or without Ta/T1
